# Tetrathiafulvalene chemistry

**DOI:** 10.3762/bjoc.11.167

**Published:** 2015-09-01

**Authors:** Peter J Skabara, Marc Sallé

**Affiliations:** 1 WestCHEM, Department of Pure and Applied Chemistry, University of Strathclyde, Glasgow G1 1XL, United Kingdom; 2 MOLTECH-Anjou, Université d’Angers, UMR CNRS 6200, 2 Bd Lavoisier, 49045 Angers Cedex, France

**Keywords:** tetrathiafulvalene, TTF

Tetrathiafulvalene (TTF) is a fascinating system: it is quite rare to find a synthetic molecule endowed with such a simple architecture that is capable of concentrating intense interest from various communities of chemists! This modest-sized molecule which consists of only 14 atoms, was synthesized in the early nineteen seventies [1–3] and since then has proved to be exceptionally popular in various fields of chemistry.

This success results from the conjunction of intrinsic structural and electronic properties: i) structurally, this sulfur-rich bicyclic compound is essentially planar (at least in the radical cation state) and therefore presents, as with most of its substituted derivatives, a good propensity to stack in the solid state. This parameter is favorable for efficient charge delocalization in the solid-state and it is this feature that gave birth to the first conducting and superconducting organic salts (organic metals); the topic of organic conductors remains a very active area of research, attested by thousands of papers from chemists and physicists interested in the transport properties of TTF-based materials [4–5]; ii) this non-aromatic 14 π-electron system is readily oxidized through a reversible process to the corresponding aromatic cation radical and dication species, according to two successive one-electron redox processes. This excellent π-donating ability, coupled to the high stability of both oxidized species, has contributed to the establishment of this unit as a key contemporary building block for the construction of redox-active molecular and supramolecular systems, where an electron-donating capacity is needed and/or when a redox-control has to be considered. It is therefore not surprising that a myriad of TTF derivatives has been produced for various applications. From that point of view, the contrast between the simplicity of the C_6_S_4_ active framework on one hand and the huge variety of topics that are encompassed by those derivatives is totally incredible. This has been possible thanks to the continuous, creative efforts developed by organic chemists to decorate this unit for specific uses; our special issue in the *Beilstein Journal of Organic Chemistry* Thematic Series presents a snap shot of this highly dynamic field.

A wide community of readers, from synthetic chemists to materials science experts, will find exciting reports (letters, full papers and reviews) spanning the most recent trends from internationally leading groups in this field. The selection of works cover a broad variety of fields that will hopefully serve to inspire the reader, implementing further research into TTF derivatives towards new horizons.

As the guest editors of this Thematic Series in the *Beilstein Journal of Organic Chemistry*, we warmly acknowledge all the authors for their outstanding contributions and the staff of the Beilstein-Institut for their excellent and professional support.

Peter Skabara and Marc Sallé

Glasgow, Angers, August 2015
